# Big Data Clustering via Community Detection and Hyperbolic Network Embedding in IoT Applications

**DOI:** 10.3390/s18041205

**Published:** 2018-04-15

**Authors:** Vasileios Karyotis, Konstantinos Tsitseklis, Konstantinos Sotiropoulos, Symeon Papavassiliou

**Affiliations:** 1 Institute of Communication and Computer Systems (ICCS), School of Electrical and Computer Engineering, National Technical University of Athens (NTUA), Athens 157 80, Greece; ktsitseklis@netmode.ntua.gr (K.T.); ksotirop@bu.edu (K.S.); papavass@mail.ntua.gr (S.P.); 2 Department of Computer Science, Boston University, Boston, MA 02215, USA

**Keywords:** data clustering, community detection, Girvan–Newman algorithm, hyperbolic network embedding, Rigel embedding, edge-betweenness centrality, smart-cities/buildings

## Abstract

In this paper, we present a novel data clustering framework for big sensory data produced by IoT applications. Based on a network representation of the relations among multi-dimensional data, data clustering is mapped to node clustering over the produced data graphs. To address the potential very large scale of such datasets/graphs that test the limits of state-of-the-art approaches, we map the problem of data clustering to a community detection one over the corresponding data graphs. Specifically, we propose a novel computational approach for enhancing the traditional Girvan–Newman (GN) community detection algorithm via hyperbolic network embedding. The data dependency graph is embedded in the hyperbolic space via Rigel embedding, allowing more efficient computation of edge-betweenness centrality needed in the GN algorithm. This allows for more efficient clustering of the nodes of the data graph in terms of modularity, without sacrificing considerable accuracy. In order to study the operation of our approach with respect to enhancing GN community detection, we employ various representative types of artificial complex networks, such as scale-free, small-world and random geometric topologies, and frequently-employed benchmark datasets for demonstrating its efficacy in terms of data clustering via community detection. Furthermore, we provide a proof-of-concept evaluation by applying the proposed framework over multi-dimensional datasets obtained from an operational smart-city/building IoT infrastructure provided by the Federated Interoperable Semantic IoT/cloud Testbeds and Applications (FIESTA-IoT) testbed federation. It is shown that the proposed framework can be indeed used for community detection/data clustering and exploited in various other IoT applications, such as performing more energy-efficient smart-city/building sensing.

## 1. Introduction

Sensor networks in future smart-cities/buildings will be larger and more heterogeneous, collecting information from radically different services and forming more complex topologies. In the advent of the IoT era, such massive, and typically distributed, sensor networks are expected to generate very large volumes of diverse types of data, raising the bar for processing efficiency and potential exploitation of such datasets. Analytics for such datasets have already become challenging, requiring further enhancements of the available techniques and at various capacities in order to sustain the anticipated scales of operation and the associated requirements.

The existing and future IoT sensor topologies will generate different measurements at faster rates, a tendency that is expected to become more demanding. Such data are cumulatively denoted as “big sensory data” [1,2]. Similarly, the computation of several graph analysis metrics that are extensively used in the study of complex topologies (e.g., Edge-Betweenness Centrality (EBC)) and overlay applications (e.g., recommendation, privacy and trust establishment systems), in an efficient and accurate manner, can be considered from a “big network data” analysis framework [3] perspective. In the latter, both large network topologies and large datasets are required to be analyzed efficiently.

Big sensory data, such as those obtained in smart cities/building networks [4], vary in volume, type and time scale, frequently forming multi-dimensional datasets, where co-located measurements of diverse types constitute complex data of multiple dimensions. Furthermore, these datasets traditionally exhibit various forms of redundancy. In order to palliate such trends, several data clustering approaches [5] have been proposed, aiming at increasing the speed and accuracy of the analysis of the data. However, as the volume of the generated sensor measurements increases at unprecedented scales, sometimes in the order of petabytes [6], data clustering techniques will require fundamental enhancements to ensure their sustainability. Various directions for this have been recently pinpointed. Among others, assuming a network representation of the data under analysis that depicts their interrelations allows one to address data clustering as a node clustering problem of the corresponding data graph. In turn, network clustering techniques such as community detection [7] can be employed, creating an alternative substrate to address data clustering. Relevant attempts include [1,2]. However, the anticipated scales of big sensory data call for further enhancements of the existing community detection approaches, ensuring proper accuracy and scaling, especially for data clustering.

In this paper, we aim towards more efficient big sensory data analytics. We propose a modification of the Girvan–Newman (GN) community detection algorithm [8] via hyperbolic network embedding, making it suitable for big sensory data clustering. This enhancement of GN is based on a new approximation approach for the computation of EBC, where node distances are computed in a graph embedded in hyperbolic space. We denote the corresponding metric as Hyperbolic Edge-Betweenness Centrality (HEBC) and modify the core idea of GN to compute HEBC rather than EBC to increase the speed of community computation and the scaling potential for cases of very large datasets without sacrificing considerable accuracy. Combining the computation of HEBC with an approach of removing up to a certain number of edges (called a batch) before recomputing the embedding used in the modified GN, we increase the speed of clustering and in certain cases the associated accuracy (in terms of the modularity measure) compared to the original GN algorithm. We studied the performance of our approach for various types of artificial benchmark datasets and networks, including topologies from real social networks, thus showing that the hyperbolic GN method can be indeed used for community detection and data clustering in various scenarios. Furthermore, we applied our scheme over real datasets obtained from the Federated Interoperable Semantic IoT/cloud Testbeds and Applications (FIESTA-IoT) platform consisting of various multi-dimensional measurements from smart-city/building sensors, such as temperature, sensor battery level, etc. We demonstrate the efficacy of our approach in such a real operational scenario and its potential benefits.

The rest of this paper has the following organization. Section 2 presents various relevant works already available in the literature and distinguishes the unique contribution of our work, while Section 3 describes our enhancement of the GN community detection algorithm via hyperbolic network embedding. Section 4 explains the proposed data clustering scheme, while Section 5 provides evaluation results over artificial datasets, benchmark topologies and real datasets. Finally, Section 6 recapitulates the essence of the proposed framework and outlines some potential directions for further investigation.

## 2. Related Work and Contribution

### 2.1. Background and Related Work

A frequently-used definition of data clustering considers it as the unsupervised classification of various observations (data) into specific groups [5]. It emerges as a suitable approach for classifying big sensor data and potentially discovering hidden correlations among them. Data clustering has been applied in various diverse applications. Examples include recommender systems where users are grouped according to their preferences [9] and healthcare applications classifying patients according to their medical history [10]. There is a great variety of general-purpose clustering algorithms [5], broadly segregated as centralized and distributed approaches. Centralized approaches may include partitioning (e.g., *k*-means [11], CLARANS [12]), hierarchical (e.g., BIRCH [13], Chameleon [14]), grid (e.g., STING [15], WaveCluster [16]) or density methods (e.g., DBSCAN [17]), among others. Centralized approaches are usually simple to implement, but suffer from scaling issues with modern big sensory datasets. Distributed methods mainly implement MapReduce or some variation of parallel clustering. MapReduce approaches are modified versions of centralized algorithms like *k*-means [18], MR-DBSCAN [19], DBCURE-MR [20], etc. Parallel clustering methods [21] typically consist of modified centralized algorithms, aiming at distributing their execution over multiple machines.

Relations between multi-dimensional or other types of data observations can be represented via a data graph, where data correspond to nodes and links between them represent their inter-relations. Data clustering using the data graph model resembles node clustering emerging in community detection in networks. The latter has become a prominent field of cross-disciplinary research. Three main classes of methodologies can be identified, namely division based, label propagation based and modularity optimization. The first suggests removing edges of the analyzed network according to specific rules, resulting in various connected components, each corresponding to a different community. The most popular representative of this category is the Girvan–Newman (GN) algorithm, which removes edges according to the EBC metric. The main observation exploited by the GN approach is that the number of edges connecting members within a community (intra-edges) is significantly greater than the number of edges connecting nodes belonging in different communities (inter-edges). These inter-community edges demonstrate a bridge-like property, and therefore, they tend to have large EBC values. Thus, removing such edges will lead to revealing the emerging communities in the network. The GN algorithm exhibits slow computational speed, since the process of EBC computation-ordering-removal of high-EBC value edges has a significant cost, and it needs to be repeatedly applied. Furthermore, in GN, the number of targeted communities is pre-specified. Modularity optimization methods typically exhibit better performance when the number of communities is unknown. Modularity is a measure used to evaluate the partition of a network in communities. It quantifies the number of edges that exist inside the communities relative to a random distribution of the total number of edges over the network. The mathematical expression of modularity is: $Q=\frac{1}{2m}\sum_{l=1}^{k}\sum_{i\in C_{l},j\in C_{l}}\left(A_{ij}-\frac{d_{i}d_{j}}{2m}\right)$, where *m* is the total number of edges in the network, *k* is the number of communities, $C_{l}$ denotes the set of nodes in the *l*-th community, A is the adjacency matrix from which $A_{ij}$ is obtained, corresponding to the actual number of edges between nodes i,j, and $d_{i}$ is the degree of node *i*. A large modularity score, where Q∈[−1,1], indicates well-connected communities, meaning that the edges inside the communities are more than those connecting different ones. Modularity optimization methods aim to find the partition of the network into communities that maximizes the total modularity score, summed over all communities. Since the problem of determining the best partition according to modularity is an NP-hard problem [22], methods aiming at Modularity Maximization (MM) apply heuristics to achieve communities, each yielding individually high modularity score and, thus, cumulatively, a high modularity value, in a reasonable amount of time.

At the same time, computing network metrics in large graphs, such as the length of shortest paths between node pairs or the EBC values needed in GN, is rather costly. By embedding the network in a low dimensional space via assigning each node coordinates can aid in accomplishing the computations more efficiently. The hyperbolic space is known to be a suitable choice when it comes to large graphs that represent various complex/social networks. This is because such graphs have been conjectured to have an underlying hyperbolic geometry [23]. There are several approaches for hyperbolic network embedding in the literature [24], such as Rigel [25], greedy [24] and Hypermap [26]. In this paper, we rely on Rigel embedding. The latter maps the network graph in hyperbolic space via multi-dimensional scaling. Rigel assumes the hyperboloid model of the *n*-dimensional hyperbolic space [25]. In this model of hyperbolic geometry, the distance between two points with coordinates $x=(x_{0},\ldots ,x_{n})$, $y=(y_{0},\ldots ,y_{n})$, is given by:

(1) $$\cosh d_{H}\left(x,y\right)=\sqrt{\left(1+{\left||x|\right|}^{2}\right)\left(1+{\left||y|\right|}^{2}\right)}-<x,y>,$$

In the above distance formula, ||·|| is the Euclidean norm and <·,·> the inner product. The solutions ($d_{H}\left(x,y\right)$) of Equation (1) are denoted as hyperbolic distances in the hyperboloid model.

In brief, Rigel operates as follows. Assume a network with *N* nodes. Rigel defines a special subset of L<<N nodes, termed landmarks, which are used as reference points. The bootstrapping step computes the proper hyperbolic coordinates of each landmark as solutions of a global optimization problem, in which the distances for all pairs of landmarks in the hyperboloid match as closely as possible their corresponding distances in the original graph measured in hops. The hyperbolic coordinates of the rest of the nodes are computed according to the coordinates of the landmarks, hence their name, so that each node’s hyperbolic distances to all landmarks are as close as possible to their corresponding hop distances in the original graph. The selection of landmarks is key, and several strategies for appropriate selection of landmark nodes are provided in [27]. Furthermore, the accuracy of Rigel increases as the dimension of hyperbolic space increases. The number of landmarks should be equal to, or even higher than the dimension of the embedding space [25]. Consequently, avoiding the computation of shortest paths for all possible node pairs and leveraging on Rigel’s properties reduce the time complexity to simple algebraic calculations of distance. This allows for faster computation of HEBC, yielding faster community detection.

### 2.2. Contribution

In this paper, we introduce a novel framework for performing data clustering via community detection. We identify the shortcomings of division-based community detection approaches, such as GN, for the purpose of data clustering at large scales and suggest an enhancement that allows the GN to run faster without significant loss of accuracy. Our proposed approach, denoted as hyperbolic GN, belongs to the class of centralized clustering techniques and aims at addressing explicitly the scaling issues emerging in other conventional centralized methods. For the first time, we suggest using hyperbolic network embedding for performing the computations required in GN more efficiently and enabling the use of this approach for big sensory data clustering, as well. We employ Rigel embedding and show that this type of embedding is rather suitable when the data graph is of a scale-free structure [28], allowing one to achieve higher accuracy than the traditional GN algorithm. This makes the proposed framework rather appealing for the cases where the data graph exhibits power-law features and other scale-free-like properties. By employing the modularity measure as a benchmark, we show the efficacy of our approach using both artificial datasets and real networks/datasets.

The contributions of this paper can be summarized as follows:

- We enhance the GN community detection algorithm through hyperbolic network embedding and by computing the HEBC of the embedded data graph, making it faster without significant accuracy loss in terms of modularity, achieving even better results in some cases.
- We facilitate the use of the enhanced GN approach over very large networks and pinpoint its potentials in various types of complex topologies representing communities or data dependency graphs.
- We introduce a framework for data clustering of big sensory data via the enhanced GN community approach.
- We demonstrate the feasibility and performance potentials of the proposed framework with benchmark and real datasets from the FIESTA-IoT testbed federation.

## 3. Community Detection Enhancement via Hyperbolic Network Embedding

Our framework for data clustering via community detection capitalizes on the Girvan–Newman (GN) approach. It enhances it with hyperbolic network embedding of the associated data graph, in order to speed-up computations and allow scaling in very large/dense data graphs. In this section, we present the proposed enhancement of GN, while in the next, we incorporate the enhanced GN in a broader framework for big sensor data clustering and parameter estimation.

### 3.1. Hyperbolic Edge-Betweenness Centrality

The original GN algorithm initially specifies the expected number of communities to be discovered. Then, the edges of the graph are ranked according to their Edge-Betweenness Centrality (EBC) value. The edge with the highest EBC value is removed from the graph. If the graph remains connected, the previous step is repeated until the graph becomes disconnected. The process repeats by examining the edges of the largest connected component. The algorithm completes when the number of connected components matches the number of communities specified in the first step.

In the GN algorithm, the computation of EBC is typically rather costly. To alleviate this burden, we suggest a new measure approximating EBC, which capitalizes on hyperbolic network embedding and can be considered as the “hyperbolic” analog of EBC. This measure is denoted as Hyperbolic Edge Betweenness Centrality (HEBC), and it is computed by utilizing the hyperbolic node coordinates assigned to the embedded nodes. Similarly to EBC, HEBC refers to each edge of the network and quantifies the number of greedy paths between any pair of nodes passing over the specific edge over the total number of such greedy paths, for all node pairs. This computation is faster than the one proposed by Brandes [29], while bearing the cost of not being 100% accurate. For the computation of HEBC, we modify the algorithm for the computation of Hyperbolic Betweenness Centrality (HBC) described in [30]. Algorithm 1 implements the new HEBC computation in pseudocode. The Supplementary Material contains links to the source code of the key functions.

For the computation of HEBC, one needs to account for the number of shortest paths crossing each edge of the data graph. In Line 2 of Algorithm 1, an outer loop sets each node as a potential destination. Inside this loop, in Part I, the nodes are sorted in a non-increasing order according to their hyperbolic distance from the node that is considered to be the destination. In this way, nodes can be examined in the correct order in the following parts. In Part II, the number of greedy paths between each node acting as source and each destination is calculated. A greedy path is a path constructed by moving from a node to a destination choosing each time a node nearest to the final destination [31]. Using greedy forwarding is another novelty of the proposed approach for speeding up the computation. Greedy forwarding using hyperbolic coordinates (in our case obtained by Rigel embedding) is known to produce greedy paths with a length close to the shortest path length [26]. In the last part of the algorithm, the dependencies δ, namely the number of greedy paths towards the destination that pass through the other nodes, are calculated for every node of the graph and so is HEBC for every edge that appears on a greedy path towards the destination node. Finally, when the outer loop concludes, the value of HEBC of every edge has been calculated.

It must be noted that the number of greedy paths is not always the same as the number of the actual shortest paths between a pair of nodes. This is the reason that the HEBC value of an edge differs from the nominal EBC value that would be computed accurately in the original graph. Furthermore, as the number of greedy paths between a node *i* and a node *j* differ from the number of greedy paths from node *j* to node *i*, the value $HEBC(u_{i},u_{j})$ would differ from the value $HEBC(u_{j},u_{i})$. In this paper, we consider that the full betweenness centrality of an edge (u,v) is the sum of the betweenness centralities of the directed edges (u,v) and (v,u). In any case, in terms of accuracy, the ranking of edges according to HEBC is very close to the ranking according to EBC, which is the desired outcome for the operation of HGN.

**Algorithm 1:** Hyperbolic Edge Betweenness Centrality (HEBC).
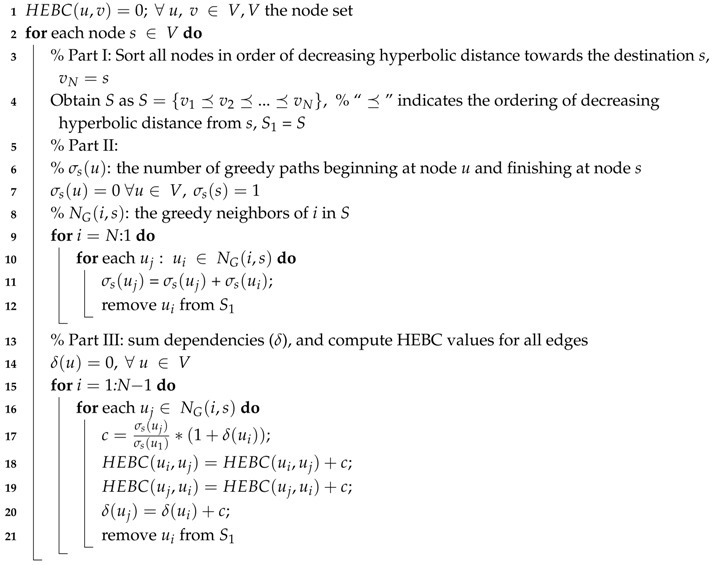


### 3.2. Hyperbolic Girvan–Newman for Community Detection

**Figure 1:**
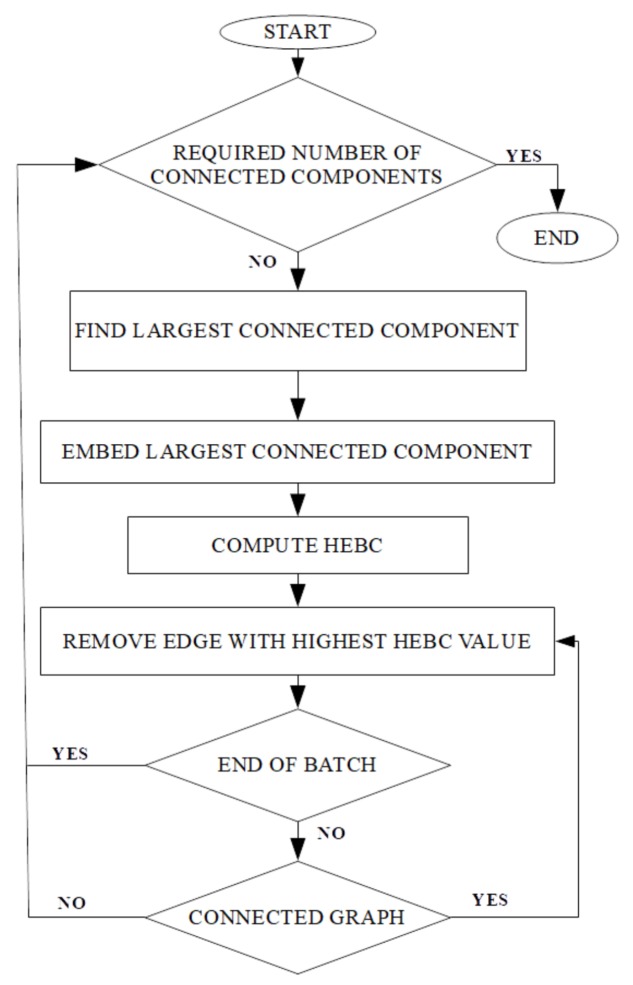
Hyperbolic Girvan–Newman algorithm.

Our proposed approach develops a modified community detection algorithm, denoted as Hyperbolic Girvan–Newman (HGN). HGN can be used for pure community detection over, e.g., social networks, or over data graphs with the penultimate goal of data clustering. In HGN, the graph is embedded in the hyperbolic space using Rigel embedding [25] with a small number of landmarks. The landmarks serve the purpose of anchor nodes, according to which the distances of all the rest of the nodes in the embedding are determined. The distances among all landmarks are computed with higher accuracy than the distances of the rest of the nodes from the landmarks. A small number of landmarks, in the range of 6–10, is adopted in this work, following similar practices as in [30]. The next step employs Algorithm 1 to calculate the HEBC value of each edge. Based on the intuition that an edge with a high EBC value is likely to act as a “bridge-like” edge, thus joining two potential communities/clusters, and that such edges are a small fraction of the total, a fixed number of edges with the highest HEBC values, called the batch, is determined. A suitable batch size *b* depends heavily on the total number of edges in the graph. Then, instead of removing a single edge at a time, as is the case with the original GN algorithm, a number of top-ranked edges equal to the batch is removed as follows. Either the batch runs out of edges and the graph remains connected, or the graph becomes disconnected. Subsequently, the largest connected component of the graph is embedded in the hyperbolic space, and the same steps are repeated until the graph is split into the required number of connected components, each of which corresponds to a different community. The intuition behind a batch is that by removing possibly more than one edge at a time, the critical edges will be removed sooner, leading to faster community detection. However, this comes at the cost of some penalty on the accuracy of our scheme, since some of the removed edges might not be the right ones to be removed. Figure 1 provides a flowchart of the HGN algorithm.

## 4. Big Sensor Data Clustering

One of the most challenging characteristics of data obtained from large sensor network topologies, such as the FIESTA-IoT smart-city/building platform [4], is the existence of multi-dimensional observations, namely measurements of various types, e.g., temperature, humidity, battery level, etc., collected at the same location by a single or multiple co-located sensors. We refer to each different type of measurement as a feature. Thus, a feature can be any type of measurement produced by the sensors, e.g., temperature, humidity, soil moisture, etc., and the number of features *f* is equal to the dimension of the multi-dimensional dataset considered in the general case. The sensors collect observations that potentially consist of a large number of features. Each observation $X_{i}\left(t\right)$ is described by its features, $X_{i}\left(t\right)={\left[X_{i}^{\left(1\right)}\left(t\right),X_{i}^{\left(2\right)}\left(t\right),X_{i}^{\left(3\right)}\left(t\right),\ldots ,X_{i}^{\left(f\right)}\left(t\right)\right]}^{T}$, where *f* denotes the dimension, i.e., number of types of different measurements collected at time moment *t*. Each component $X_{i}^{\left(j\right)}\left(t\right)$ of an observation *i* is a sample of the corresponding feature *j*, e.g., temperature, at time *t*. Let $X\left(t\right)=\left\{X_{1}\left(t\right),X_{2}\left(t\right),X_{3}\left(t\right),\ldots ,X_{n}\left(t\right)\right\}$ be the set of data/observations produced by a multi-dimensional sensor network at time *t*, consisting of observations $X_{i}\left(t\right)$, where *n* is the number of sensors of the network.

Each observation $X_{i}\left(t\right)$ of the dataset can be perceived as a point in a Euclidean space of size *f*. A very large number of features *f* probably will impose problems in the analysis of the dataset in terms of execution time and scaling. This problem, often referred to as the “curse of dimensionality”, can be treated by dimensionality reduction techniques [32]. It is essential for these techniques to maintain useful information, such as which observations are similar to each other. In this context, we employ a graph representation of data, which allows maintaining those relations among data that are considered valuable, suppressing the rest of the information.

Graphs that are constructed by linking observations in a metric space using a distance metric are called proximity graphs [33]. A careful selection of a methodology for the formation of the proximity graph, as well as the choice of a suitable community detection algorithm leads to communities that resemble a natural clustering of the original data points. To construct the proximity graph, we employ a method that makes use of minimum spanning trees in the graph. The approach employed in this work is the one based on Disjoint Minimum Spanning Trees (DMST), described in [34]. This method yields a graph that is the union of the first *k* minimum spanning trees, where *k* is a parameter defined by the user. As mentioned in [34], a relatively small *k* is sufficient for the graph to capture the spatial information of the dataset with a standard number of edges. This approach outweighs the ϵ-ball approach [34], which heavily relies on the choice of radius ϵ, denoting the maximum distance between two connected nodes.

Before community detection is applied, the initial dataset is cleaned, and any observations with “out-of-range” or missing values are removed. Then, every observation is considered as a node in a metric space with dimensions equal to the features of each observation, and the full weighted graph is produced. Each edge of the graph has weight equal to the distance of the nodes it joins. Then, the DMST graph is produced from the full graph for a small number of trees. This graph is then embedded in the hyperbolic space using the Rigel embedding, and finally, the communities are produced by the HGN algorithm with the appropriate selection of the batch size and the number of communities to be discovered.

## 5. Evaluation

In this section, we evaluate the proposed framework with artificial and real datasets. Section 5 is divided into three subsections. In the first, results regarding the computation of HEBC for real and artificial topologies are provided. The second focuses on the evaluation of the proposed data clustering approach with respect to the involved parameters over various benchmark and real datasets. Finally, the third part investigates the application of the proposed methodology on datasets obtained from operational large-scale smart-city/building sensor networks, obtained from the FIESTA-IoT testbed. For the performed analyses, a desktop with Intel Core i5-4570 3.20 GHz CPU, 8 GB RAM (Intel, Santa Clara, CA, USA) and Windows 10 (64 bit) OS (Microsoft, Seattle, WA, USA) was used for executing all computations.

### 5.1. Evaluation and Performance Assessment of HEBC Computation

**Figure 2:**
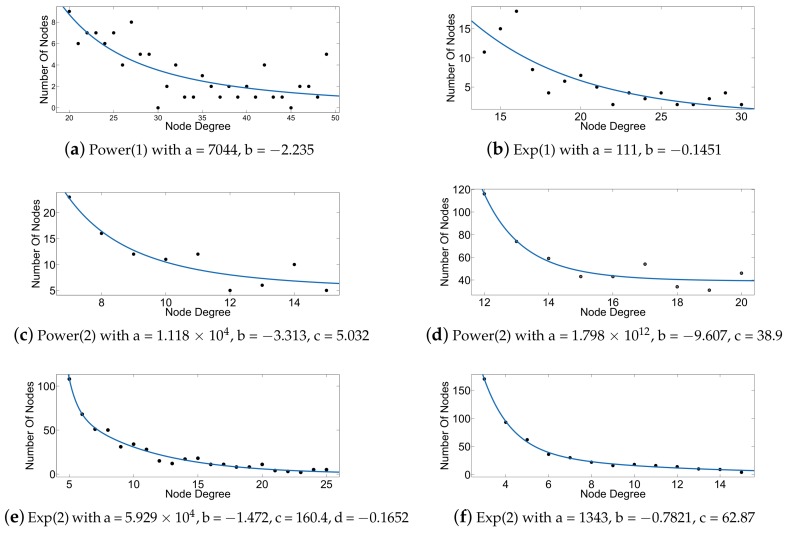
Curve fitting for the degree distribution of artificial networks. (**a**) Dense100; (**b**) Mid100; (**c**) Sparse100; (**d**) Dense500; (**e**) Mid500; (**f**) Sparse500. The parameters provided in each panel’s caption correspond to the fitting models summarized in Table 1.

**Figure 3:**
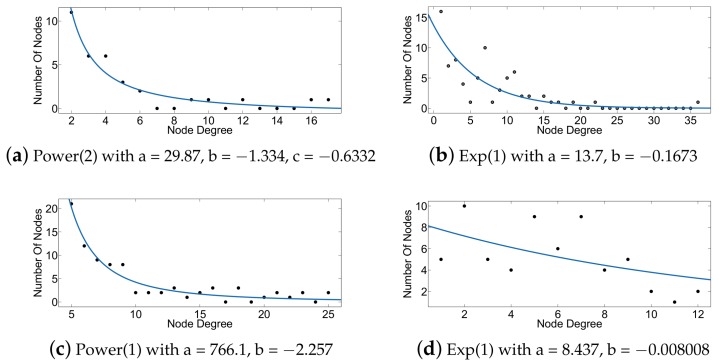
Curve fitting for degree distribution of real networks. (**a**) Karate; (**b**) lesmis; (**c**) polbooks; (**d**) dolphins. The parameters provided in each panel’s caption correspond to the fitting models summarized in Table 1.

In this subsection, we present the accuracy of computing HEBC metric in large scale-free artificial networks and some real social networks obtained from [35]. We have analyzed the degree distribution of the employed networks. Table 1 shows the form of the fitting models we obtained, while Figure 2 and Figure 3 show the curve fitting over the degree distributions for the artificial and real networks employed in the following evaluations of our approach. Knowledge of the actual structure of each network allows better explanation of the benefits of our approach.

**Figure 4:**
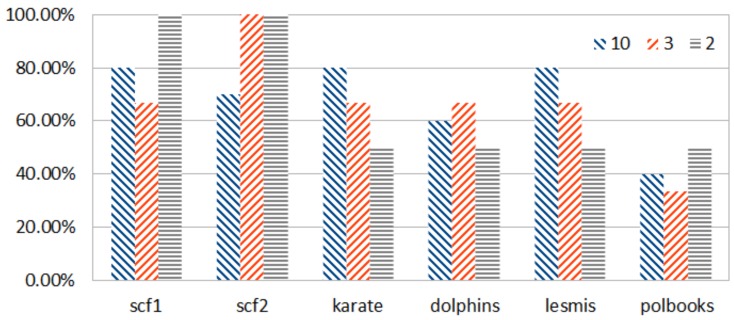
Percentage of correct prediction in the top-*k* ranked edges for the networks presented in Table 2. Blue columns denote the accuracy achieved for the top 10 (first of each triplet of bars), red for the top *k* = 3 (second of each triplet of bars) and yellow for the top *k* = 2 edges (third of each triplet of bars).

Figure 4 presents the percentage of correct prediction of the top-*k* edges ranked according to EBC and HEBC and shows that notable scores of accuracy are achieved for these types of scale-free and exponential topologies. EBC ranking is used as a reference. Figure 4 quantifies the percentage of accuracy achieved by the ranking according to HEBC with respect to the ranking of the top-*k* edges by EBC. This happens because using Rigel embedding for power-law and power-law-like (exponential) networks yields less distortion of the distances in the embedded network, i.e., they are closer to the original ones. The satisfactory results for the social networks examined are justified, since such networks are known to follow a power-law or a power-law-like degree distribution [28].

In Table 2, the HEBC metric is compared to the EBC value obtained by Brandes’ computation, described in [29], in terms of execution time. Although the time needed for the completion of HEBC value is less for every benchmark graph, the unavoidable overhead imposed by the embedding makes the HEBC algorithm practically faster for large graphs with more than a few hundred nodes, as shown in the last column of Table 2.

By the last observation, a pattern emerges indicating that each approach is suitable for a different range with respect to the size of the data graph. GN appears more appropriate for smaller sizes, while HGN for the larger topologies. In the sequel, in Section 5.2.2, we provide additional relevant results, which not only demonstrate the effectiveness of HGN in very large topologies, but also delimit the more appropriate operational scales for each scheme (GN-HGN).

### 5.2. Data Clustering Framework via Hyperbolic Network Embedding

#### 5.2.1. Known Communities

**Figure 5:**
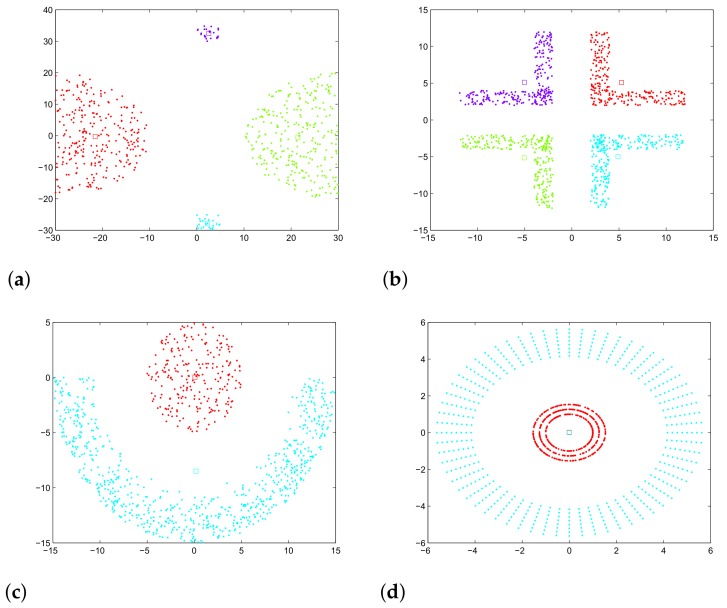
Artificial datasets employed. (**a**) Outliers; (**b**) corners; (**c**) fullmoon; (**d**) cluster in cluster. The colors denote the discovered communities, demonstrating 100% accuracy.

To evaluate the performance and accuracy of the proposed algorithm for clustering via community detection, a number of graphs was utilized. Initially, we examined proximity graphs, which were generated from artificial 2-dimensional datasets. In this case, community detection can be verified visually (known ground truth). The employed datasets can be viewed in Figure 5 (consisting of all data points), over which the HGN algorithm detected communities (the discovered communities are shown using different colors for the data points). In addition, benchmark graphs for testing the GN algorithm were used, produced by the algorithm described in detail in [36]. For these graphs, the communities are known beforehand (ground truth), so a direct evaluation of the proposed algorithm’s accuracy is possible based on the modularity measure, and this is shown in the following parts of the paper. Information about these graphs is provided in Table 3.

The effect of the batch size on the execution time of the algorithm is important. In Table 4 and Table 5, we present the effect of the batch size on the clustering time required over the proximity graphs. For brevity, only the configurations that result in 100% success are presented. From these tables, it can be seen that, in most cases, as the batch size increases, the execution time decreases. This behavior is expected because a bigger batch size ensures that more edges are removed from the graph before the re-embedding and the HEBC phases begin. As the embedding phase is the most time-consuming part of the algorithm, fewer embeddings yield faster execution times. At the same time, one must be careful with the batch size, because a large value will drive the algorithm to produce faster results, possibly resulting in a penalty on the accuracy, since removing many edges at a time may involve removing edges that would not be removed by the original GN algorithm in that order.

**Figure 6:**
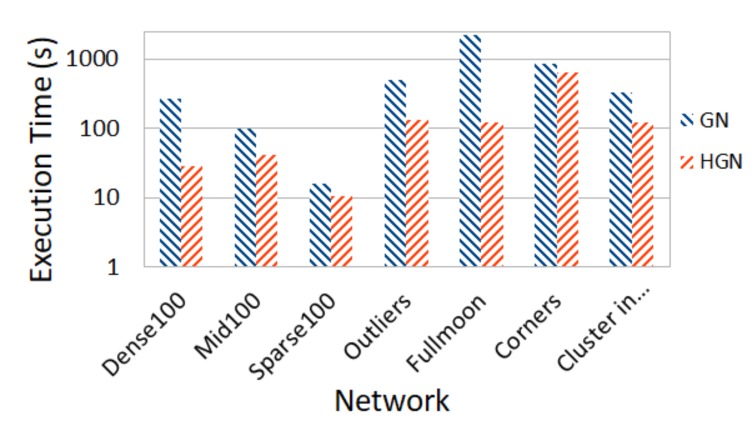
Execution time of GN and HGN algorithms for graphs with known communities (logarithmic scale).

Furthermore, we compare the execution time of the GN and HGN algorithms. In Table 6, the execution time for each of the graphs of Table 3 is presented. It must be noted that with the exception of two graphs, namely Dense500 and Mid500, HGN yielded 100% accurate results for all the other graphs. For the two graphs mentioned before, in the case of Dense500, only two nodes are assigned wrongly, while in the case of Mid500, 50% accuracy was achieved. It is noteworthy to mention that for the outlier graph, the traditional GN algorithm fails to discover correctly the four communities shown in Figure 5, while the HGN algorithm succeeds. Figure 6 provides the information of Table 6 visually for the graphs for which HGN correctly discovered their communities.

#### 5.2.2. Unknown Communities

Except from networks with distinct and known communities, we examine networks for which the ground truth is unknown. To assess the level of accuracy achieved, we employ the Modularity Maximization (MM) method, as described in [37]. The application of the MM algorithm produces a partition of nodes in modules (communities), assigning a modularity score to each community. The higher the modularity score, which ranges in [−1, 1], the better the community partition according to this measure. With respect to the modularity measure, MM achieves the maximum score obtained for any partition of the given nodes (data points), serving as a benchmark of the best achievable community partition in the following evaluations. Both the classic GN and the HGN algorithms are executed with input the number of communities equal to the number found by MM. The intuition behind this comes from the fact that if MM achieves the best possible partition according to the modularity measure, the other approaches must yield at least the same number of communities in order to approach the modularity score achieved by MM. Their partitions into communities are assessed by computing the corresponding cumulative modularity value.

The batch size is chosen in each case to be the one that produces the best modularity scores. For this evaluation, we employ two different types of graphs, namely scale-free (scf) and random geometric (rgg), representative of the relational and spatial graph paradigms, respectively [28]. We use artificial and real topologies. For the first, the Barabasi-Albert method was employed [38]. The basic characteristics of the artificial networks used for this evaluation are presented in Table 7 for the scale-free topologies and Table 8 for the random geometric ones, respectively. Similar features were introduced for the real social networks previously employed in Section 4.

As seen in Table 9, the GN algorithm completes in most cases faster than the HGN, but HGN achieves better modularity scores, corresponding to better community detection (clustering accuracy). However, it should be noted that many of these graphs are relatively small in size. As the size increases, the computational efficiency of HGN emerges. Regarding the cases in which the produced modularities differ significantly, this is due to the fact that in these topologies, the edges that connect low degree nodes to nodes of high degree have high EBC values. Eventually, these edges are removed from the graph. This leads to the formation of a large community accompanied by very small communities of a few nodes, thus explaining the low modularity score achieved by the GN algorithm. Table 10 shows the size of the communities formed by the GN algorithm, applied over scale-free graphs. It can be seen that for every network, almost all the communities, except for the one discovered by the HGN algorithm, have at most four nodes. Moreover, these nodes have a low degree, and this can be verified by examining the mean degree of the nodes composing each community. This verifies our hypothesis for the formation of one “giant” community accompanied by many small ones. On the other hand, since the HEBC accuracy for these topologies is higher, this means that different edges were removed. These were edges that connect nodes of high degree between them, leading to the formation of communities that result in greater modularity scores.

**Figure 7:**
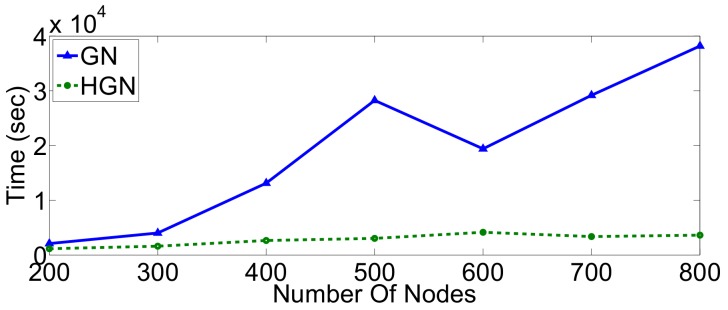
Execution time of the GN and HGN algorithms in scale-free networks.

**Figure 8:**
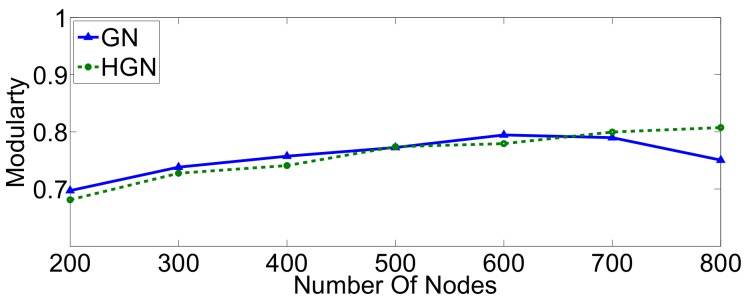
Modularity scores achieved by GN and HGN in scale-free networks.

To further investigate the performance differences with respect to execution time and modularity score (accuracy) between the HGN and GN approaches at larger scales, we consider topologies of increasing cardinality (number of nodes) for two different network types, namely small-world and random geometric. Firstly, a number of small-world graphs was generated with nodes ranging from 200–800. As can be confirmed from Figure 7, there exists a threshold around 300 nodes, beyond which the HGN algorithm terminates significantly faster, while achieving good results of modularity, relatively close to those of the GN algorithm (Figure 8). Furthermore, for these types of graphs, and especially for very large topologies, the accuracy of HGN can be greater than that achieved by GN with respect to the modularity metric. This strengthens our assumption that one can safely use the proposed method, saving time without compromising the involved accuracy considerably.

**Figure 9:**
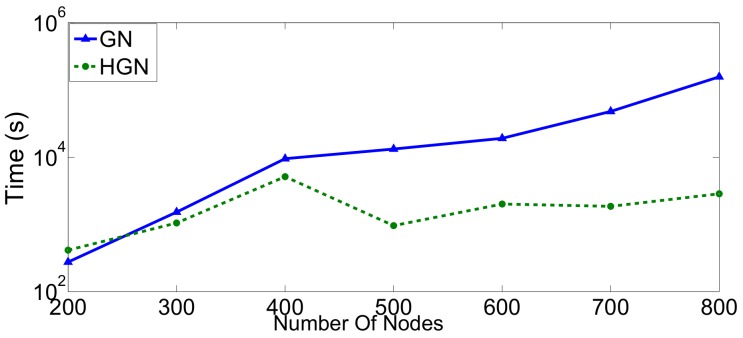
Execution time of the GN and HGN algorithms in random geometric networks.

**Figure 10:**
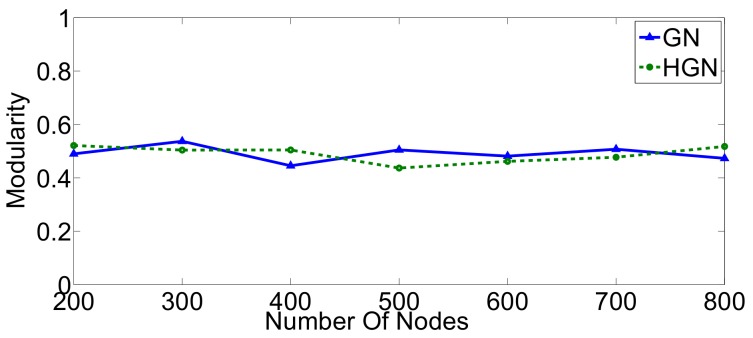
Modularity scores achieved by GN and HGN in random geometric networks.

We have repeated this evaluation for random geometric graphs in the same range of 200–800 nodes. The results are provided in Figure 9 and Figure 10. Similarly to the case of small-world graphs, our algorithm completed its execution faster, especially for larger topologies (Figure 9). The threshold above which HGN executes significantly faster than GN is now 400 nodes. The accuracy performance measured in terms of modularity is comparable for both approaches (Figure 10). Consequently, even though HGN offers the same fidelity, its computational performance makes it more appropriate for big sensor data environments, ensuring the required scaling in these cases.

We should stress that the number of edges in the above random geometric and small-world topologies ranged in the order of thousands, e.g., 3000–4000 edges for a typical scenario. In real scenarios, the number of edges may grow even faster, signifying the importance of good computational scaling in these operational ranges. From the above results, HGN shows remarkable scaling adaptation in terms of computational time, without considerable accuracy penalty, constituting a very suitable alternative for big sensor data analytics.

### 5.3. Real Evaluation on FIESTA-IoT Datasets

In this subsection, we evaluate the proposed clustering framework over data graphs constructed from real dataset, which were obtained from the FIESTA-IoT federation and more specifically the SmartSantander testbed [39]. This testbed allowed us to collect large sets of multi-dimensional data, reaching up to approximately 500 nodes. Each node corresponds to co-located sensor data for various features, such as GPS coordinates, temperature, battery levels, etc. It should be highlighted that SmartSantander is among the very few testbeds allowing the collection of that many multi-dimensional data (up to 500 distinct co-located sensor points), uniquely allowing a realistic evaluation of HGN that is closer to the anticipated big sensor data scales of the future.

The datasets were obtained at different sampling instances, using different sampling rates, e.g., 1 sample/5 min, 1 sample/20 min, etc. After the raw data were obtained, a cleaning process followed to ensure that any missing or damaged data were removed. Then, data from co-located sensors (i.e., sharing the same latitude and longitude values) were considered as multi-dimensional data and treated as described in Section 4. We were able to collect around 500 distinct multi-dimensional data points at each time. Hence, for each sampling instance, the corresponding data graph of around 500 nodes was obtained and embedded in the hyperbolic space. Then, the HGN algorithm was applied in order to produce data clusters corresponding to specific sampling instances. We compared the performance of our scheme against the other two (GN, MM) employed before in terms of the modularity metric.

**Figure 11:**
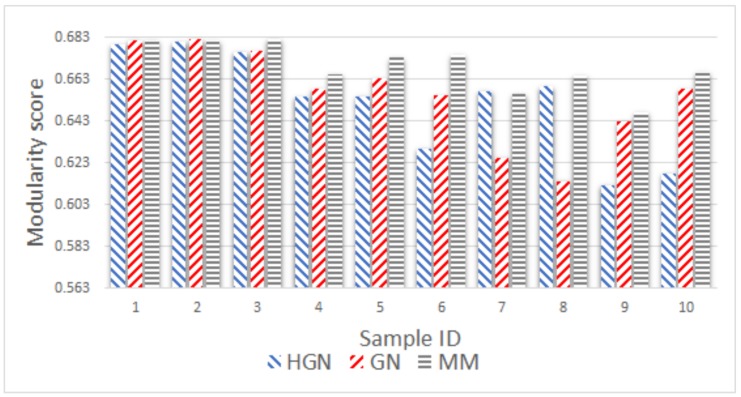
Modularity comparison/5 min sampling (15 December 2017, 11:00–12:00).

**Figure 12:**
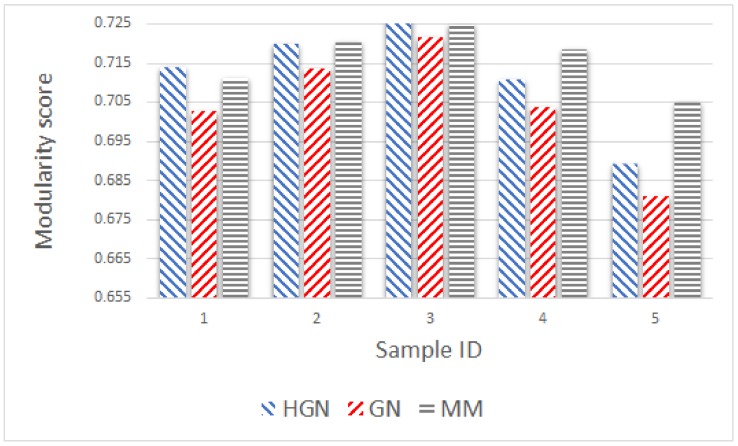
Modularity comparison/10 min sampling (15 December 2017, 11:00–12:00).

**Figure 13:**
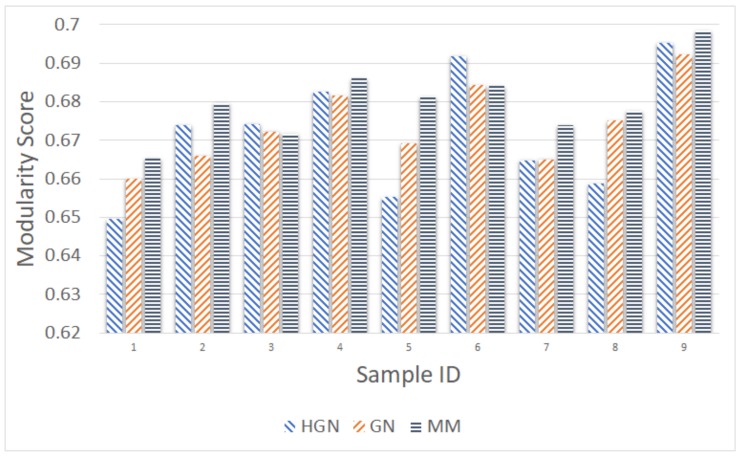
Modularity comparison/20 min sampling (15 December 2017, 11:00–13:00).

**Figure 14:**
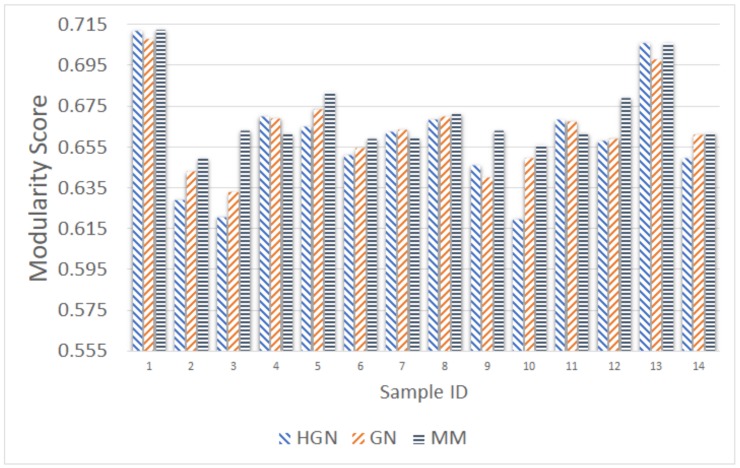
Modularity comparison/30 min sampling (15 December 2017, 11:00–18:00).

**Figure 15:**
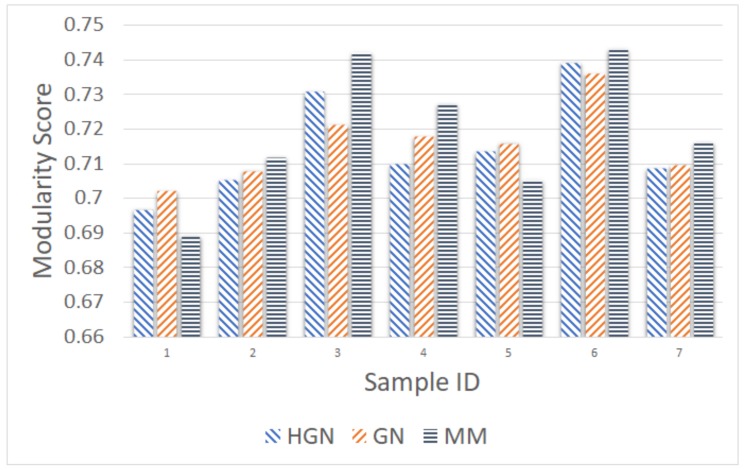
Modularity comparison/60 min sampling (15 December 2017, 11:00–18:00).

As before, the comparison took place using as input the number of communities resulting from the MM approach. Figure 11, Figure 12, Figure 13, Figure 14 and Figure 15 present the modularity score obtained for each of the obtained community clusterings. Each figure corresponds to different sampling rates of the testbeds’ resources. Each sample in each figure corresponds to a distinct proximity graph. Taking into account the scale of the modularity score in each data series (different sampling rate), it can be deduced that all three approaches are relatively close, yielding data clusters that in turn yield similar scores with respect to the modularity metric employed for their evaluation. Thus, the proposed HGN approach can match closely the accuracy of the conventional GN and MM approaches, sacrificing a very small percentage of it. In fact, the corresponding performance can be attained for any sampling rate employed, signifying the practical potentials of HGN in various energy-efficiency smart-city/building applications.

Combined with analogous results for artificial networks and benchmark datasets provided earlier in Section 5.2.2, we can conclude that the proposed methodology can be safely employed for data clustering in unknown and operational scenarios, expecting approximately the same accuracy level as with previously employed approaches (GN, MM), while offering more computational efficiency over GN.

## 6. Conclusions

In this paper, we proposed a novel data clustering approach for measurements obtained from large IoT sensor topologies. Starting with a graph representing data dependencies, we suggested embedding it in the hyperbolic space and modified the Girvan–Newman community detection algorithm so that the edge-betweenness centrality of each link required for community detection is computed faster in the hyperbolic space and sometimes with better accuracy. We demonstrated the potentials of the proposed approach in terms of scaling and computational efficiency with synthetic and real datasets. We showed that the proposed framework can yield accurate results for datasets that potentially exhibit very big data scales and quantified its performance against typically employed benchmark approaches.

Our future work will focus on developing an adaptive approach for determining the optimal batch size and the optimization of the parameters of Rigel embedding, pertinent to the type and scale of the involved data graph. Furthermore, we will apply the HGN approach in an energy-efficient clustering application over FIESTA-IoT, exploiting it to reduce the number of sensors needed at each time in indoor and outdoor environments, thus reducing the associated energy cost while maintaining monitoring accuracy.

## Figures and Tables

**Table 1:** Fitting models for the degree distribution of the employed networks.

Model	Expression
Exp(1)	$a*e^{b*x}$
Exp(2)	$a*e^{b*x}+c*e^{d*x}$
Power(1)	$a*x^{b}$
Power(2)	$a*x^{b}+c$

**Table 2:** Time comparison (in sec.) between Brandes’ EBC and HEBC.

Networks	Nodes	Edges	EBC	HEBC	Rigel (Landmarks)	HEBC + Rigel (Total) Time
scf1	1000	5788	39.03	4.07	18.10 (15)	22.17
scf2	1000	5821	121.76	3.84	8.41 (15)	12.25
karate	34	78	0.03	0.01	9.68 (15)	9.69
dolphins	62	166	0.09	0.02	4.28 (10)	4.30
lesmis	77	258	0.15	0.04	5.87 (10)	5.91
polbooks	105	442	0.31	0.04	20 (15)	20.04

**Table 3:** Characteristics of the GN benchmark graphs.

Network	Nodes	Edges	Mean Degree	Max. Degree	Communities
Dense100	100	1493	29.86	49	4
Mid100	100	954	19.08	30	3
Sparse100	100	496	9.92	15	4
Dense500	500	3781	15.12	30	7
Mid500	500	2398	9.59	25	7
Sparse500	500	1389	5.55	15	7

**Table 4:** Effect of batch size on the execution time (in sec.) of the HGN algorithm for the proximity graphs. Dashes denote results that were not possible to obtain with the respective batch size.

Batch Size	Outliers	Corners	Fullmoon	Cluster in Cluster
25	-	512.36	187.28	111.5
50	195.39	233.40	119.25	107.37
100	58.89	-	236.02	79.62
200	-	-	-	119.34
500	-	-	-	61.64
1000	-	-	-	34.88

**Table 5:** Effect of batch size on the execution time (in sec.) of the HGN algorithm for the benchmark graphs. Dashes denote results that were not possible to obtain with the respective batch size.

Batch Size	Dense100	Mid100	Sparse100
10	78.48	46.54	9.69
20	55.45	25.04	8.02
30	44.52	23.08	6.64
40	40.34	16.77	-
50	35.53	-	6.44
60	32.04	-	6.67
70	28.56	-	-

**Table 6:** Comparison of execution time (in sec.) of GN and HGN algorithms.

Network	GN Time	HGN Time (Batch Size)
Dense100	236.93	28.63 (60)
Mid100	86.18	40.78 (10)
Sparse100	13.86	10.62 (10)
Dense500	9634.99	3754.02 (10)
Mid500	2838.15	2575.79 (5)
Sparse500	2752.45	3221.47 (2)
outliers	416.69	130.41 (50)
fullmoon	1048.82	119.25 (50)
corners	442.29	233.40 (50)
cluster in cluster	227.27	120.70 (100)

**Table 7:** Characteristics of the employed scale-free networks.

Network	Nodes	Edges	Min. Degree	Max. Modularity
scf3	100	356	4	0.31
scf4	100	427	5	0.27
scf5	100	557	7	0.22

**Table 8:** Characteristics of random geometric networks.

Network	Nodes	Edges	Threshold	Max Modul
rgg1	100	612	0.2	0.65
rgg2	100	1152	0.3	0.51
rgg3	100	1732	0.4	0.40
rgg4	100	2394	0.5	0.30

**Table 9:** Modularity and time (in sec.) comparison of HGN and GN.

Network	GN Time	Modularity of GN	HGN Time	Modularity of HGN
karate	0.54	0.34	14.14	0.42
dolphins	1.92	0.36	29.99	0.45
lesmis	6.42	0.44	52.10	0.55
polbooks	15.17	0.51	42.85	0.51
scf3	37.52	0.096	384.14	0.28
scf4	44.92	0.0031	659.30	0.21
scf5	79.02	−0.00032	1229.35	−0.062
rgg1	15.23	0.63	19.74	0.63
rgg2	98.31	0.49	34.17	0.51
rgg3	31.68	0.00017	56.89	0.40
rgg4	141.45	−0.00068	174.97	0.28

**Table 10:** Mean node degree of each community after GN clustering. The size of each community is given in the parentheses, i.e., single, double or quad node communities. The first three topologies have 7 communities, while the fourth 8.

Topology	Scf3	Scf4	Scf5	Scf6
Community 1	5 (1)	5 (2)	4 (1)	5 (1)
Community 2	4 (1)	5.25 (4)	4 (1)	5 (1)
Community 3	4 (1)	5.6 (2)	4 (1)	6 (1)
Community 4	6 (1)	4.33 (4)	5 (2)	6 (1)
Community 5	7 (1)	4.5 (2)	4 (1)	6 (1)
Community 6	10.67 (94)	7.61 (80)	8.85 (93)	6 (1)
Community 7	7 (1)	5.25 (4)	5 (1)	11.55 (93)
Community 8	-	-	-	6 (1)
Mean Network Degree	10.04	7.12	8.54	11.14

## Supplementary Materials

The basic functions for the computation of HEBC are available online at: https://github.com/netmode/CREDIT_FIESTA-IoT_OC1_experiment.

## Abbreviations

The following abbreviations are used in this manuscript:

DMST	Disjoint Minimum Spanning Trees
EBC	Edge-Betweenness Centrality
GN	Girvan–Newman
HBC	Hyperbolic Betweenness Centrality
HEBC	Hyperbolic Edge-Betweenness Centrality
HGN	Hyperbolic Girvan–Newman
MM	Modularity Maximization
RGG	Random Geometric Graph
SCF	Scale-free
